# Weight stigma and mental health in a racially and ethnically diverse sample of US adults

**DOI:** 10.3389/fpsyt.2025.1593145

**Published:** 2025-07-21

**Authors:** Mary A. Gerend, Anna W. Lu, Elizabeth L. Teets

**Affiliations:** College of Medicine, Florida State University, Tallahassee, FL, United States

**Keywords:** weight stigma, perceived weight discrimination, internalized weight bias, global mental health, depressive symptoms, depression, adaptive coping strategies

## Abstract

**Introduction:**

Weight stigma is associated with poor mental health outcomes. Yet little is known about whether the strength of the association between weight stigma and mental health outcomes differs by race or ethnicity, or factors that mitigate the mental health consequences of weight stigma. This study sought to address these research gaps.

**Methods:**

A large sample of US adults (N = 2,632; aged 18–64 years; 50% women) completed an online survey. Quota sampling ensured that over two-thirds of respondents self-identified as Black/African American or Hispanic/Latino. Our primary predictors were experienced and internalized weight stigma. Primary outcomes included global mental health, depression severity, and history of diagnosis with a depressive disorder. Linear and logistic multivariable regression analyses tested whether the association between weight stigma and mental health outcomes was moderated by (1) race or ethnicity, and (2) frequency of using adaptive coping strategies to manage weight stigma-related stress (e.g., cognitive reframing, seeking social support).

**Results:**

Both experienced and internalized weight stigma were associated with worse mental health (i.e., lower global mental health scores, more frequent depressive symptoms in the past two weeks, greater odds of depressive disorder diagnosis) and effects held while controlling for body mass index and sociodemographic characteristics. Further, the strength of the association between weight stigma and mental health outcomes was equivalent among Black and non-Black participants and among Latino and non-Latino participants. Adaptive coping was a significant moderator for global mental health and depressive disorder diagnosis but not depression severity such that the weight stigma-mental health relationship was weakest among respondents who engaged in adaptive coping strategies more frequently.

**Discussion:**

Adults with more frequent exposure to interpersonal weight stigma and higher levels of internalized weight bias reported poorer mental health status. Notably, the strength of these associations was similar regardless of racial or ethnic identity suggesting no group is protected from the detrimental health effects associated with weight stigma. Individuals who respond to weight stigma with adaptive coping strategies may be more protected from adverse psychological outcomes. Findings have important implications for initiatives aimed at reducing harm to mental health that may be associated with weight stigma.

## Introduction

People with high body weight are devalued in American society. Being devalued because of one’s body weight is known as *weight stigma* and can be a powerful source of chronic stress (1). Weight stigma arises from the inaccurate belief that body weight is highly controllable, leading to the perception that individuals are personally responsible for their weight (2). Indeed, people with high body weight are stereotyped as lazy, incompetent, and lacking in self-discipline (1, 3). Furthermore, these negative stereotypes are often used to justify discriminatory behavior against individuals with high body weight (2). Weight-based discrimination frequently occurs during interpersonal interactions with strangers, service employees, health care providers, and family members (4). It can also manifest as environmental barriers that disadvantage people with higher body weight or larger body size. Examples include inadequate medical equipment in clinics or hospitals, or poorly designed seating in public spaces (5). Repeated exposure to weight discrimination along with awareness of negative weight-based stereotypes also contributes to the internalization of weight stigma such that individuals come to devalue themselves because of their body weight (2, 6–8). Considerable evidence shows that weight stigma—whether self-directed or external—is a common experience for individuals with high body weight; approximately 20-40% have been exposed to weight stigmatizing experiences in their lifetime and over 50% endorse high levels of internalized weight stigma (9, 10).

Research consistently demonstrates a negative association between weight stigma and mental health outcomes (11–13). A meta-analysis of over 100 studies with nearly 60,000 participants confirmed that weight stigma is associated with worse mental health outcomes (*r* = -0.35; moderate effect size) (14). While the strength of the association varied across outcomes (*r* = -0.22 to -0.39), weight stigma was associated with a variety of mental health conditions including dysfunctional and disordered eating behavior, body image dissatisfaction, psychological distress, low self-esteem, and symptoms of depression or anxiety (14). Further, this negative association was observed for both experienced weight stigma (*r* = -0.33; also referred to as *perceived weight discrimination*) and internalized weight stigma (*r* = -0.39; also referred to as *internalized weight bias*).

Theoretical models describing the relationship between discrimination and health conceptualize weight stigma as a chronic stressor that impairs health through psychological, behavioral, and physiological pathways (2, 15–19). With respect to psychological pathways, weight stigma may contribute to poor mental health outcomes via increases in negative affect or use of poor emotion regulation strategies in response to discriminatory experiences (e.g., rumination, inability to reframe negative thoughts) (20–22). Weight stigma also triggers a variety of behaviors that can be harmful for mental wellbeing including emotional (“comfort”) eating, sleep disturbance, substance use, and withdrawal from social activities (5, 19, 23–27). Indeed, some of these psychological and behavioral responses may reflect coping strategies aimed at reducing the immediate emotional burden of weight stigma (28). Physiologically, exposure to weight stigma has been shown to increase cardiovascular reactivity, secretion of cortisol, and inflammation (29–32). Over time, repeated activation of these pathways can impair mental health.

Despite the large body of research on weight stigma and mental health, relatively few studies have examined this association in Black and Latino populations. This gap is noteworthy, as Black and Latino individuals living in the United States face multiple forms of stigma and discrimination in their day-to-day lives (33, 34). Emmer and colleagues (14) conducted an exploratory analysis to test ethnicity as a potential moderator of the association between weight stigma and mental health outcomes. They found no evidence, however, that the strength of the relationship between weight stigma and mental health outcomes differed between White and non-White individuals. Nevertheless, an important limitation of this research was the fact that individuals from different cultural backgrounds were combined into a single category (i.e., “non-White”) due to the low representation of Black and Latino individuals in previous weight stigma studies. Such an approach could obscure potential differences. To examine the independent contribution of both race and ethnicity as potential moderators of the link between weight stigma and mental health, the current study intentionally oversampled adults who self-identified as African American or Black and/or Hispanic or Latino. This was done to ensure sufficient representation of individuals from groups that are disproportionately affected by cultural stigma and discrimination in the US.

Further, surprisingly little is known about factors that mitigate the negative relationship between weight stigma and mental health (14). Such work is critical for informing future interventions aimed at reducing the harmful mental health consequences associated with weight stigma. Some research suggests that the coping strategies people use to manage weight stigma-related stress could have important implications for mental health (22, 28, 35, 36). For example, individuals who seek social support from family or friends after a weight stigmatizing encounter may be buffered from its negative psychological effects. Likewise, responding to weight stigma with cognitive reappraisal tactics (e.g., reframing an interpersonal encounter with weight discrimination as reflecting the perpetrator’s own insecurity and low-self-esteem) may weaken the impact of weight stigma on mental health.

The aims of the present study were twofold. The first aim was to assess whether the strength of the association between weight stigma (experienced and internalized) and mental health outcomes varies by race or ethnicity. Due to limited research in this area, we did not have specific predictions about the moderating effects of race or ethnicity. The second aim was to assess whether the strength of the association between weight stigma and mental health outcomes depends on the coping strategies people use to manage stress from weight stigma. More specifically, we hypothesized that more frequent use of adaptive coping strategies (e.g., seeking support, using cognitive reframing, practicing self-acceptance) would weaken the association between weight stigma and mental health outcomes. We recruited a large sample of US adults with a broad representation across the body mass index (BMI) spectrum and a disproportionately high proportion of respondents who self-identified as Black or African American and/or Hispanic or Latino. We examined the association between weight stigma and three mental health outcomes in particular: self-reported global mental health, severity of depressive symptoms over the past two weeks, and previous diagnosis with a depressive disorder.

## Materials and methods

### Participants and procedure

This study was approved by the Institutional Review Board at Florida State University. All respondents provided electronic informed consent before they could begin the survey. Data for this study were drawn from a large cross-sectional study on weight stigma and health that was collected using Dynata’s online sampling platform. A more detailed description of the study procedure is provided elsewhere (25). Potential respondents received a notification announcing the study opportunity, along with the estimated time to complete the survey (10–15 minutes) and a pre-determined incentive that would be awarded upon completion. To be eligible for the study, respondents had to be US residents between the ages of 18 to 64 years, have a BMI value between 12 to 70 kg/m^2^, self-identify as a cisgender man or woman, and self-identify as Black or African American, Hispanic or Latino, or non-Hispanic White. The sample size goal for the study was 2,500 respondents. Quotas for race and ethnicity were specified in advance to oversample respondents who self-identified as Black or African American (≥33% of the sample) and/or Hispanic or Latino (≥33% of the sample). To further increase the diversity of the sample, we also oversampled respondents with a non-heterosexual sexual orientation (e.g., self-identified as gay, lesbian, or bisexual; ≥25% of the sample). Quotas were not specified for BMI. A total of 3,028 respondents completed the survey; however, 396 respondents were excluded for data quality concerns (e.g., failing attention checks, completing the survey in <30% of the median time). The final sample size for analysis was 2,632 respondents.

### Measures

#### Weight stigma

Predictor variables of interest were experienced and internalized weight stigma. Experienced weight stigma was assessed with the Stigmatizing Situations Survey-Brief (SSI-B) (37), a 10-item version of the original 50-item scale (38). Respondents rated how often they experienced ten different “situations that people encounter because of their weight.” Sample items include: “Having a doctor recommend a diet even if you did not come in to discuss weight loss” and “Overhearing other people making rude remarks about you in public.” Following Puhl and Brownell (39), respondents rated how often they experienced each situation in their lifetime using a 4-point scale: 0 = *never*; 1 = *once*; 2 = *more than once*; 3 = *multiple times*. Items were averaged to create a total score representing lifetime experience with weight stigma. SSI-B scores ranged from 0 to 3, with higher scores indicating more frequent experience with weight stigma (Cronbach’s α = .92 for the current sample).

Internalized weight stigma was assessed with the Modified Weight Bias Internalization Scale (WBIS-M) (8). Respondents rated their agreement with 11 items using a 7-point scale that ranged from 1 = *strongly disagree* to 7 = *strongly agree*. Sample items include: “I am less attractive than most other people because of my weight” and “Whenever I think a lot about my weight, I feel depressed.” Following previous research (10, 21, 40), we excluded one reverse-scored item (i.e., “Because of my weight, I feel that I am just as competent as anyone.”) that often has poor psychometric properties. The remaining 10 items were averaged to create a total score. WBIS-M scores ranged from 1 to 7, with higher scores indicating higher levels of internalized weight stigma (Cronbach’s α = .93 for the current sample).

#### Mental health variables

Primary outcome variables were global mental health, depression severity, and diagnosis with a depressive disorder. Global mental health was assessed with two items from the Patient Reported Outcomes Measurement and Information System [PROMIS^®^] project (41): (1) “In general, how would you rate your mental health, including your mood and your ability to think?” and (2) “In general, how would you rate your satisfaction with your social activities and relationships?” Items were rated on a 5-point scale: 1 = *poor*; 2 = *fair*; 3 = *good*; 4 = *very good*; 5 = *excellent*. The two items were averaged to create a total score. Scores ranged from 1 to 5 with higher scores representing better global mental health. Depression severity was assessed with the two-item version of the Patient Health Questionnaire (PHQ-2) (42). Respondents indicated how often they had been bothered by any of the following problems over the last two weeks: (1) “Little interest or pleasure in doing things.” and (2) “Feeling down, depressed, or hopeless.” Items were rated on a 4-point scale: 0 = *not at all*; 2 = *several days*; 3 = *more than half the days*; 4 = *nearly every day*. The two items were summed to create a total score. Scores ranged from 0 to 6 with higher scores representing higher severity of depressive symptoms. Previous diagnosis with a depressive disorder was assessed with a single item from the Behavioral Risk Factor Surveillance System (BRFSS) (43): “Has a doctor, nurse, or other health professional ever told you that you had a depressive disorder (including depression, major depression, dysthymia, or minor depression)?” Respondents who selected “yes” received a score of 1 while respondents who selected “no” received a score of 0.

#### Adaptive coping

Adaptive coping was assessed with six items we created for this study. We chose to create our own items for the study given limitations of existing measures (i.e., too lengthy or limited in scope) (20, 38). Items drew on our previous qualitative research that identified common strategies used to manage weight stigma-related stress (28), as well as the Coping Responses Inventory (38) and previous research on coping with racism (44). (Please see supplemental materials for an exploratory factor analysis of the coping items. Only those items that clearly loaded on the adaptive coping factor were included.) Coping strategies were assessed among the subset of respondents who indicated they had ever been teased, treated unfairly, or discriminated against because of their weight and/or attributed experiences with everyday discrimination to their weight (n = 1,546). Participants were asked “When you are teased, treated unfairly, or discriminated against because of your weight, how often do you do any of the following things in response? (1) Talk to other people about it; (2) Speak up for yourself; (3) See it as their problem not yours; (4) Work harder to prove them wrong; (5) Think about your good qualities; and (6) Love and accept yourself even when it seems like other people don’t.” Items were rated on a 5-point scale: 1 = *never*; 2 = *rarely*; 3 = *every now and then*; 4 = *often*; 5 = *very often*. The six items were averaged to create a total score. Scores ranged from 1 to 5 with higher scores representing higher frequency of using adaptive coping strategies to manage weight stigma-related stress (Cronbach’s α = .79 for the current sample).

#### Demographic characteristics and body mass index

Sociodemographic characteristics assessed included age, sex, gender, ethnicity, race, sexual orientation, highest level of education, and annual household income. Respondents were asked to indicate their current height and weight which was used to compute BMI (kg/m^2^).

### Statistical analysis

Descriptive statistics were computed for sample characteristics, experienced and internalized weight stigma, and mental health outcome variables. Correlations among predictor and outcome variables were also estimated. We used linear and logistic multivariable regression to predict each outcome variable from weight stigma (experienced and internalized, assessed using separate models) while controlling for covariates. Covariates included sociodemographic characteristics and BMI. To assess whether the association between weight stigma and mental health outcomes was moderated by race (Black vs. non-Black) or ethnicity (Latino vs. non-Latino) centered interaction terms between each type of weight stigma (i.e., experienced and internalized) and race and ethnicity were added to the model. Likewise, to assess whether adaptive coping moderated the association between weight stigma and mental health outcomes, variables representing the centered interaction between each type of weight stigma and adaptive coping were added to the model. Significant interactions were followed with simple effects tests to assess the pattern of the interaction. Sensitivity power analysis (two-tailed tests with alpha set to.05) indicated that the study was adequately powered (power ≥.80) to detect effects as small as r ≥.055 across models. Given the preliminary nature of this investigation, we did not adjust our alpha levels to correct for family wise error so as to provide future researchers with greater opportunities to follow up on these findings.

Although the data were not missing completely at random (MCAR), Little’s MCAR test, χ^2^ (40) = 63.588. *p* = .010, whether a respondent was missing data on the variable responsible for the significant test (i.e., annual income, for which 73 of the 76 respondents who were missing this variable selected ‘prefer not to answer’), was not significantly correlated with any of the primary outcome variables. Respondents with lower education levels and younger age were, however, more likely to be missing a value for income. Because having a missing value for annual income was not associated with any of the outcome variables, we used listwise deletion to handle missing data in the regression analyses (45).

## Results

Sample characteristics are provided in **Table 1**. The mean age of respondents was 36.9 years (SD = 12.5). The sample included equivalent numbers of men and women. As intended, Black/African American and Hispanic/Latino participants were overrepresented in the sample with 36% of respondents identifying as Black or African American and 36% identifying as Hispanic or Latino. Eight percent (n = 212) of respondents identified as both Black/African American and Hispanic/Latino. Nearly 30% of the sample described their sexual orientation as non-heterosexual with 12% identifying as gay or lesbian and 17% identifying as bisexual, pansexual, or queer. With respect to highest level of education, 4% had less than a high school education, 28% had a high school degree or equivalent, 26% attended some college, 14% completed an associate degree or technical school, 18% had a bachelor’s degree, 8% had a master’s degree, and 2% had a doctoral degree. Median annual income fell between $35,000-$49,999. Mean BMI of the sample was 27.9 kg/m^2^ (SD = 8.2).

**Table 1:** Sample characteristics (N = 2,632).

Variable	Mean (SD) or N (%)
Age (years)	36.9 (12.5)
Gender	
Men	1327 (50)
Women	1305 (50)
Latino or Hispanic ethnicity	
No	1690 (64)
Yes	942 (36)
Black or African American race	
No	1683 (64)
Yes	949 (36)
Race	
American Indian or Alaska Native Asian Black or African American Native Hawaiian or Other Pacific Islander White Multiracial Unknown	36 (1) 10 (<1) 888 (34) 8 (<1) 1493 (57) 80 (3) 117 (4)
Sexual orientation	
Bisexual, pansexual, or queer Gay or lesbian Straight or heterosexual	454 (17) 312 (12) 1866 (71)
Annual household income	
Less than $10,000 $10,000 - $24,999 $25,000 - $34,999 $35,000 - $49,999 $50,000 - $74,999 $75,000 - $99,999 $100,000 - $149,999 $150,000 or more Prefer not to answer or unknown	306 (12) 396 (15) 374 (14) 350 (13) 463 (18) 275 (10) 238 (9) 154 (6) 76 (3)
Highest level of education	
Less than high school High school diploma/equivalent Some college Associate degree or technical school Bachelor’s degree/College graduate Master’s degree Doctoral degree	92 (4) 743 (28) 677 (26) 375 (14) 470 (18) 218 (8) 57 (2)
BMI (kg/m^2^)	27.9 (8.2)

Percentages may exceed 100% due to rounding error.

Descriptive statistics and correlations among key study variables are provided in **Table 2**. Consistent with previous studies (21), a large positive correlation was observed between experienced and internalized weight stigma (*r* = .56, *p* <.001). Ratings of global mental health fell just above a score of 3, where 3 represented “good” self-rated mental health. The mean depression severity score was 2.29, indicating that, on average, respondents experienced depressive symptoms at least “several days” over the last two weeks. Thirty-seven percent of the sample reported having a depressive disorder that had been diagnosed by a health care provider. Medium-sized correlations were observed among the three mental health variables.

**Table 2:** Descriptive statistics and correlations among experienced weight stigma, internalized weight stigma, and mental health outcomes.

Variable	n	Range	*M*	*SD*	1	2	3	4	5
1. Experienced weight stigma (SSI-B)	2620	0-3	0.83	0.83	–				
2. Internalized weight stigma (WBIS-M)	2632	1-7	3.38	1.56	.56*	–			
3. Global mental health	2629	1-5	3.11	1.14	-.16*	-.30*	–		
4. Depression severity (PHQ-2)	2627	0-6	2.29	1.95	.49*	.46*	-.46*	–	
5. Depression diagnosis	2516	0-1	0.37	0.48	.26*	.24*	-.38*	.38*	–

SSI-B, Stigmatizing Situations Survey-Brief; WBIS-M, Modified Weight Bias Internalization Scale; PHQ-2, Patient Health Questionnaire-2.

*p <.001.

Results from regression analyses that examined whether the association between weight stigma and mental health outcomes (i.e., global mental health, depression severity, and depressive disorder diagnosis) was moderated by race or ethnicity are reported in **Tables 3**–**5**, respectively. The top portion of each table presents the findings for the analysis with experienced weight stigma (i.e., SSI-B scores) as the primary predictor while the bottom portion of the table presents the findings for the analysis with internalized weight stigma (i.e., WBIS-M scores) as the primary predictor. Both experienced and internalized weight stigma were robustly associated with worse mental health as indicated by lower global mental health scores, more frequent depressive symptoms in the past two weeks, and greater odds of diagnosis with a depressive disorder. Moreover, no interactions between weight stigma and race, or between weight stigma and ethnicity were observed, indicating that the strength of the association between weight stigma (experienced and internalized) and each mental health outcome was equivalent among Black and non-Black participants, and among Latino and non-Latino participants.

**Table 3:** Linear regressions predicting global mental health from weight stigma and interactions between weight stigma and race and ethnicity.

Variable	*B*	*SE*	95% CI for *B*		*t*	*Partial r*	*p*
			*LL*	*UL*			
First set of regression findings							
SSI-B	-.190	.026	-.242	-.139	-7.208	-.142	<.001
Black	.229	.046	.139	.319	4.990	.099	<.001
Latino	.028	.047	-.063	.120	.605	.012	.545
Gender	-.252	.045	-.339	-.164	-5.651	-.112	<.001
Sexual orientation	-.246	.048	-.340	-.152	-5.131	-.102	<.001
Age	.001	.002	-.003	.005	.514	.010	.607
Income	.077	.012	.053	.102	6.256	.123	<.001
Education	.075	.017	.041	.109	4.361	.086	<.001
BMI	-.015	.003	-.021	-.010	-5.525	-.109	<.001
SSI-B x Black	-.051	.054	-.157	.055	-.938	-.019	.348
SSI-B x Latino	-.045	.054	-.150	.060	-.837	-.017	.403
Second set of regression findings							
WBIS-M	-.196	.014	-.223	-.169	-14.021	-.268	<.001
Black	.161	.045	.073	.249	3.585	.071	<.001
Latino	.032	.045	-.056	.121	.718	.014	.473
Gender	-.234	.043	-.319	-.149	-5.416	-.107	<.001
Sexual orientation	-.237	.047	-.329	-.146	-5.102	-.101	<.001
Age	-.001	.002	-.004	.003	-.510	-.010	.610
Income	.079	.012	.056	.103	6.615	.130	<.001
Education	.080	.017	.048	.113	4.811	.095	<.001
BMI	-.008	.003	-.014	-.003	-3.084	-.061	.002
WBIS-M x Black	-.013	.029	-.069	.043	-.467	-.009	.641
WBIS-M x Latino	-.047	.028	-.103	.008	-1.682	-.033	.093

*B*, unstandardized regression coefficient; *SE*, standard error; CI, confidence interval; *LL*, lower limit; *UL*, upper limit. SSI-B, Stigmatizing Situations Survey-Brief; WBIS-M, Modified Weight Bias Internalization Scale. The top portion of the table presents findings for the analysis with experienced weight stigma (i.e., SSI-B as the primary predictor) while the bottom portion of the table presents the findings for the analysis with internalized weight stigma (i.e., WBIS-M as the primary predictor). Black: 1, Black or African American race; 0, non-Black or African American race. Latino: 1, Hispanic or Latino; 0, non-Hispanic or non-Latino. Gender: 1, woman; 0, man. Sexual orientation: 1, gay, lesbian, bisexual, queer, or pansexual; 0, straight or heterosexual.

**Table 4:** Linear regressions predicting depression severity from weight stigma and interactions between weight stigma and race and ethnicity.

Variable	*B*	*SE*	95% CI for *B*		*t*	*Partial r*	*p*
			*LL*	*UL*			
First set of regression findings							
SSI-B	1.132	.042	1.051	1.214	27.168	.475	<.001
Black	-.146	.072	-.288	-.005	-2.024	-.040	.043
Latino	.004	.074	-.141	.148	.050	.001	.960
Gender	.092	.070	-.046	.230	1.311	.026	.190
Sexual orientation	.175	.076	.027	.324	2.312	.046	.021
Age	-.014	.003	-.019	-.008	-4.737	-.094	<.001
Income	-.022	.019	-.060	.017	-1.112	-.022	.266
Education	-.055	.027	-.109	-.002	-2.046	-.041	.041
BMI	-.016	.004	-.024	-.007	-3.618	-.072	<.001
SSI-B x Black	.064	.085	-.103	.231	.752	.015	.452
SSI-B x Latino	-.020	.084	-.186	.145	-.241	-.005	.809
Second set of regression findings							
WBIS-M	.582	.023	.536	.627	25.256	.448	<.001
Black	.022	.074	-.123	.167	.298	.006	.766
Latino	.058	.074	-.088	.204	.782	.016	.435
Gender	.047	.071	-.092	.186	.660	.013	.509
Sexual orientation	.224	.077	.074	.374	2.921	.058	.004
Age	-.014	.003	-.020	-.008	-4.816	-.095	<.001
Income	-.019	.020	-.058	.019	-.980	-.019	.327
Education	-.044	.027	-.098	.009	-1.615	-.032	.106
BMI	-.025	.005	-.034	-.016	-5.428	-.107	<.001
WBIS-M x Black	.002	.047	-.090	.094	.038	.001	.969
WBIS-M x Latino	.031	.046	-.060	.122	.674	.013	.500

*B*, unstandardized regression coefficient; *SE*, standard error; CI, confidence interval; *LL*, lower limit; *UL*, upper limit. SSI-B, Stigmatizing Situations Survey-Brief; WBIS-M, Modified Weight Bias Internalization Scale. The top portion of the table presents findings for the analysis with experienced weight stigma (i.e., SSI-B as the primary predictor) while the bottom portion of the table presents the findings for the analysis with internalized weight stigma (i.e., WBIS-M as the primary predictor). Black: 1, Black or African American race; 0, non-Black or African American race. Latino: 1, Hispanic or Latino; 0, non-Hispanic or non-Latino. Gender: 1, woman; 0, man. Sexual orientation: 1, gay, lesbian, bisexual, queer, or pansexual; 0, straight or heterosexual.

**Table 5:** Logistic regressions predicting diagnosis with a depressive disorder from weight stigma and interactions between weight stigma and race and ethnicity.

Variable	*B*	*SE*	Wald	*OR*	95% CI		*p*
					*LL*	*UL*	
First set of regression findings							
SSI-B	.673	.057	140.390	1.959	1.753	2.190	<.001
Black	-.394	.100	15.637	.675	.555	.820	<.001
Latino	-.088	.101	.749	.916	.751	1.118	.387
Gender	.457	.095	23.232	1.579	1.312	1.902	<.001
Sexual orientation	.682	.099	47.575	1.977	1.629	2.400	<.001
Age	.005	.004	1.614	1.005	.997	1.013	.204
Income	-.086	.027	10.321	.918	.871	.967	.001
Education	-.070	.037	3.609	.932	.867	1.002	.057
BMI	.010	.006	2.974	1.010	.999	1.022	.085
SSI-B x Black	.143	.117	1.498	1.154	.918	1.450	.221
SSI-B x Latino	.201	.117	2.960	1.222	.972	1.536	.085
Second set of regression findings							
WBIS-M	.317	.031	106.321	1.373	1.293	1.459	<.001
Black	-.271	.098	7.588	.763	.629	.925	.006
Latino	-.038	.100	.140	.963	.791	1.172	.708
Gender	.419	.093	20.135	1.520	1.266	1.826	<.001
Sexual orientation	.711	.098	52.888	2.037	1.681	2.467	<.001
Age	.004	.004	.984	1.004	.996	1.011	.321
Income	-.081	.026	9.621	.922	.876	.971	.002
Education	-.059	.037	2.581	.943	.878	1.013	.108
BMI	.006	.006	1.166	1.006	.995	1.018	.280
WBIS-M x Black	.035	.064	.299	1.035	.914	1.173	.584
WBIS-M x Latino	.064	.063	1.047	1.067	.943	1.207	.306

*B*, unstandardized regression coefficient; *SE*, standard error; *OR*, odds ratio; CI, confidence interval; *LL*, lower limit; *UL*, upper limit. SSI-B, Stigmatizing Situations Survey-Brief; WBIS-M, Modified Weight Bias Internalization Scale. The top portion of the table presents findings for the analysis with experienced weight stigma (i.e., SSI-B as the primary predictor) while the bottom portion of the table presents the findings for the analysis with internalized weight stigma (i.e., WBIS-M as the primary predictor). Black: 1, Black or African American race; 0, non-Black or African American race. Latino: 1, Hispanic or Latino; 0, non-Hispanic or non-Latino. Gender: 1, woman; 0, man. Sexual orientation: 1, gay, lesbian, bisexual, queer, or pansexual; 0, straight or heterosexual.

Results from regression analyses that examined whether the association between weight stigma and global mental health, depression severity, and depressive disorder diagnosis was moderated by adaptive coping are reported in **Tables 6**–**8**, respectively. Again, the top portion of each table represents findings for the analysis with experienced weight stigma as the primary predictor, while the bottom portion of the table represents the findings for the analysis with internalized weight stigma as the primary predictor. Adaptive coping was a significant moderator of the association between weight stigma and global mental health and between weight stigma and previous diagnosis with depression; however, adaptive coping did not moderate the association between weight stigma and depression severity.

**Table 6:** Linear regressions predicting global mental health from weight stigma and the interaction between weight stigma and adaptive coping.

Variable	*B*	*SE*	95% CI for *B*		*t*	*Partial r*	*p*
			*LL*	*UL*			
First set of regression findings							
SSI-B	-.168	.034	-.234	-.101	-4.947	-.127	<.001
Adaptive coping	.321	.032	.258	.384	9.987	.251	<.001
Black	.126	.056	.015	.236	2.235	.058	.026
Latino	-.028	.055	-.136	.079	-.514	-.013	.607
Gender	-.186	.055	-.295	-.078	-3.359	-.087	.001
Sexual orientation	-.163	.058	-.276	-.050	-2.821	-.073	.005
Age	.000	.002	-.004	.005	.119	.003	.905
Income	.068	.015	.038	.097	4.474	.115	<.001
Education	.056	.021	.015	.097	2.675	.069	.008
BMI	-.010	.003	-.016	-.004	-3.269	-.085	.001
SSI-B x Adaptive coping	.167	.032	.105	.229	5.251	.135	<.001
Second set of regression findings							
WBIS-M	-.163	.018	-.198	-.127	-9.077	-.229	<.001
Adaptive coping	.276	.031	.215	.337	8.902	.225	<.001
Black	.071	.056	-.038	.180	1.280	.033	.201
Latino	-.026	.054	-.132	.079	-.487	-.013	.627
Gender	-.192	.054	-.299	-.085	-3.518	-.091	<.001
Sexual orientation	-.165	.057	-.276	-.054	-2.910	-.075	.004
Age	-.001	.002	-.006	.003	-.579	-.015	.563
Income	.083	.015	.053	.112	5.560	.143	<.001
Education	.063	.021	.023	.104	3.056	.079	.002
BMI	-.006	.003	-.012	.000	-2.019	-.052	.044
WBIS-M x Adaptive coping	.068	.017	.034	.102	3.955	.102	<.001

*B*, unstandardized regression coefficient; *SE*, standard error; CI, confidence interval; *LL*, lower limit; *UL*, upper limit. SSI-B, Stigmatizing Situations Survey-Brief; WBIS-M, Modified Weight Bias Internalization Scale. The top portion of the table presents findings for the analysis with experienced weight stigma (i.e., SSI-B as the primary predictor) while the bottom portion of the table presents the findings for the analysis with internalized weight stigma (i.e., WBIS-M as the primary predictor). Black: 1, Black or African American race; 0, non-Black or African American race. Latino: 1, Hispanic or Latino; 0, non-Hispanic or non-Latino. Gender: 1, woman; 0, man. Sexual orientation: 1, gay, lesbian, bisexual, queer, or pansexual; 0, straight or heterosexual.

**Table 7:** Linear regressions predicting depression severity from weight stigma and the interaction between weight stigma and adaptive coping.

Variable	*B*	*SE*	95% CI for *B*		*t*	*partial r*	*p*
			*LL*	*UL*			
First set of regression findings							
SSI-B	1.043	.057	.931	1.154	18.299	.429	<.001
Adaptive coping	-.274	.054	-.380	-.168	-5.066	-.131	<.001
Black	-.080	.095	-.265	.106	-.842	-.022	.400
Latino	.008	.092	-.173	.189	.083	.002	.934
Gender	.045	.093	-.138	.228	.481	.012	.631
Sexual orientation	.053	.097	-.138	.244	.544	.014	.586
Age	-.014	.004	-.022	-.006	-3.461	-.090	.001
Income	-.004	.025	-.053	.046	-.139	-.004	.890
Education	-.033	.035	-.102	.037	-.928	-.024	.354
BMI	-.019	.005	-.029	-.009	-3.625	-.094	<.001
SSI-B x Adaptive coping	.055	.054	-.050	.160	1.034	.027	.301
Second set of regression findings							
WBIS-M	.567	.031	.507	.627	18.575	.434	<.001
Adaptive coping	.026	.053	-.077	.130	.496	.013	.620
Black	.065	.095	-.121	.251	.686	.018	.493
Latino	.085	.092	-.095	.265	.926	.024	.355
Gender	.051	.093	-.131	.233	.549	.014	.583
Sexual orientation	.107	.097	-.082	.297	1.114	.029	.266
Age	-.010	.004	-.018	-.002	-2.439	-.063	.015
Income	-.029	.025	-.079	.021	-1.146	-.030	.252
Education	-.031	.035	-.100	.038	-.870	-.023	.384
BMI	-.027	.005	-.037	-.017	-5.215	-.134	<.001
WBIS-M x Adaptive coping	.054	.029	-.004	.111	1.842	.048	.066

*B*, unstandardized regression coefficient; *SE*, standard error; CI, confidence interval; *LL*, lower limit; *UL*, upper limit. SSI-B, Stigmatizing Situations Survey-Brief; WBIS-M, Modified Weight Bias Internalization Scale. The top portion of the table presents findings for the analysis with experienced weight stigma (i.e., SSI-B as the primary predictor) while the bottom portion of the table presents the findings for the analysis with internalized weight stigma (i.e., WBIS-M as the primary predictor). Black: 1, Black or African American race; 0, non-Black or African American race. Latino: 1, Hispanic or Latino; 0, non-Hispanic or non-Latino. Gender: 1, woman; 0, man. Sexual orientation: 1, gay, lesbian, bisexual, queer, or pansexual; 0, straight or heterosexual.

**Table 8:** Logistic regressions predicting diagnosis with a depressive disorder from weight stigma and the interaction between weight stigma and adaptive coping.

Variable	*B*	*SE*	Wald	*OR*	95% CI		*p*
					*LL*	*UL*	
First set of regression findings							
SSI-B	.585	.078	56.433	1.795	1.541	2.091	<.001
Adaptive coping	-.064	.072	.796	.938	.814	1.080	.372
Black	-.240	.124	3.729	.786	.616	1.004	.053
Latino	-.004	.122	.001	.996	.784	1.265	.974
Gender	.508	.122	17.256	1.663	1.308	2.114	<.001
Sexual orientation	.660	.126	27.446	1.935	1.512	2.478	<.001
Age	.004	.005	.455	1.004	.993	1.014	.500
Income	-.062	.034	3.389	.940	.879	1.004	.066
Education	-.045	.047	.951	.956	.872	1.047	.329
BMI	.008	.007	1.543	1.008	.995	1.022	.214
SSI-B x Adaptive coping	-.145	.074	3.872	.865	.748	.999	.049
Second set of regression findings							
WBIS-M	.276	.041	45.093	1.317	1.216	1.428	<.001
Adaptive coping	.132	.072	3.382	1.141	.991	1.312	.066
Black	-.164	.124	1.736	.849	.665	1.083	.188
Latino	.038	.121	.099	1.039	.819	1.318	.753
Gender	.492	.121	16.473	1.636	1.290	2.074	<.001
Sexual orientation	.705	.125	31.862	2.023	1.584	2.584	<.001
Age	.005	.005	1.075	1.005	.995	1.016	.300
Income	-.078	.033	5.390	.925	.866	.988	.020
Education	-.041	.046	.782	.960	.877	1.051	.377
BMI	.005	.007	.533	1.005	.992	1.019	.465
WBIS-M x Adaptive coping	-.100	.040	6.155	.905	.836	.979	.013

*B*, unstandardized regression coefficient; *SE*, standard error; *OR*, odds ratio; CI, confidence interval; *LL*, lower limit; *UL*, upper limit. SSI-B, Stigmatizing Situations Survey-Brief; WBIS-M, Modified Weight Bias Internalization Scale. The top portion of the table presents findings for the analysis with experienced weight stigma (i.e., SSI-B as the primary predictor) while the bottom portion of the table presents the findings for the analysis with internalized weight stigma (i.e., WBIS-M as the primary predictor). Black: 1, Black or African American race; 0, non-Black or African American race. Latino: 1, Hispanic or Latino; 0, non-Hispanic or non-Latino. Gender: 1, woman; 0, man. Sexual orientation: 1, gay, lesbian, bisexual, queer, or pansexual; 0, straight or heterosexual.

**Figure 1:**
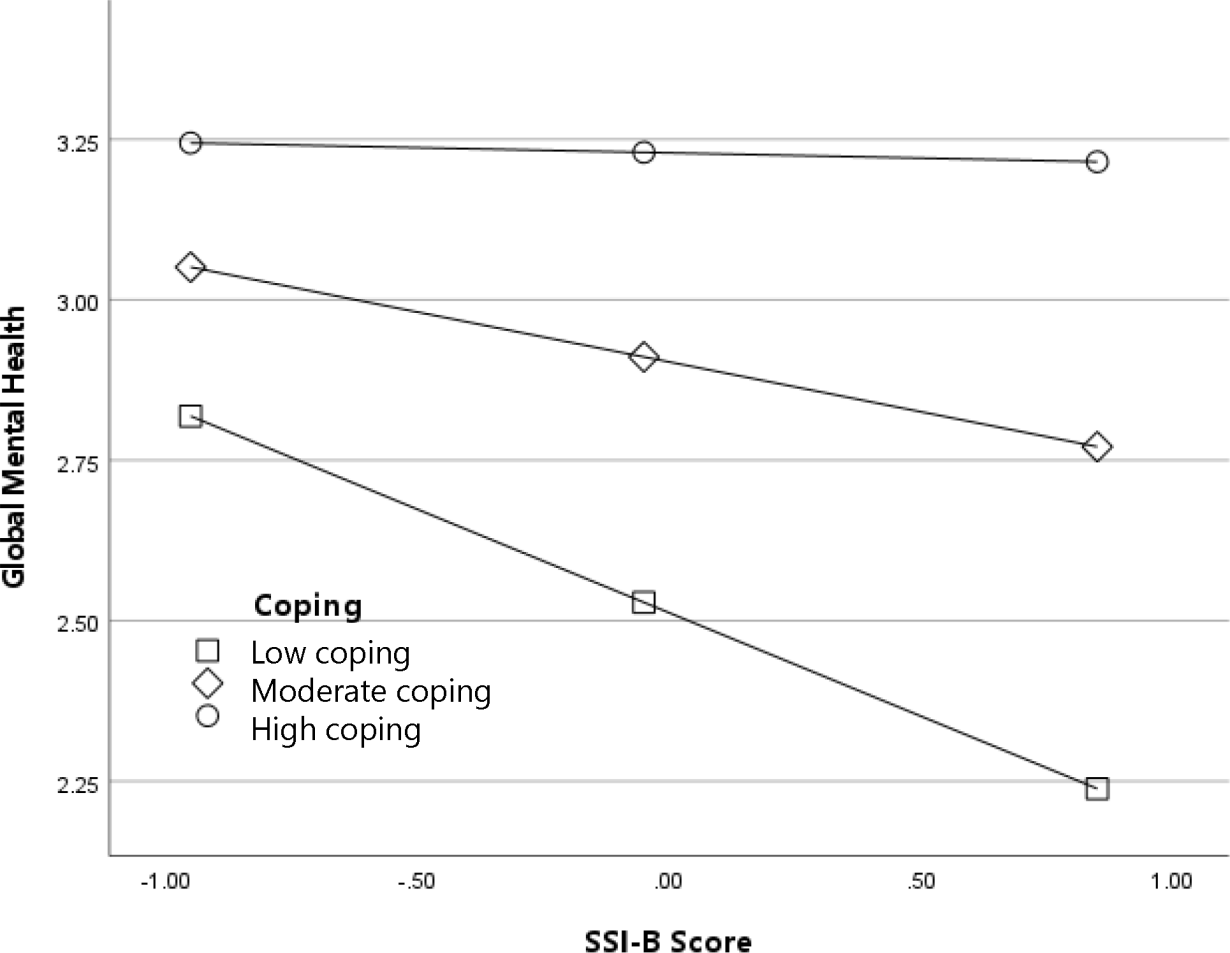
Adaptive coping as a moderator of the association between experienced weight stigma (SSI-B scores) and global mental health.

**Figure 2:**
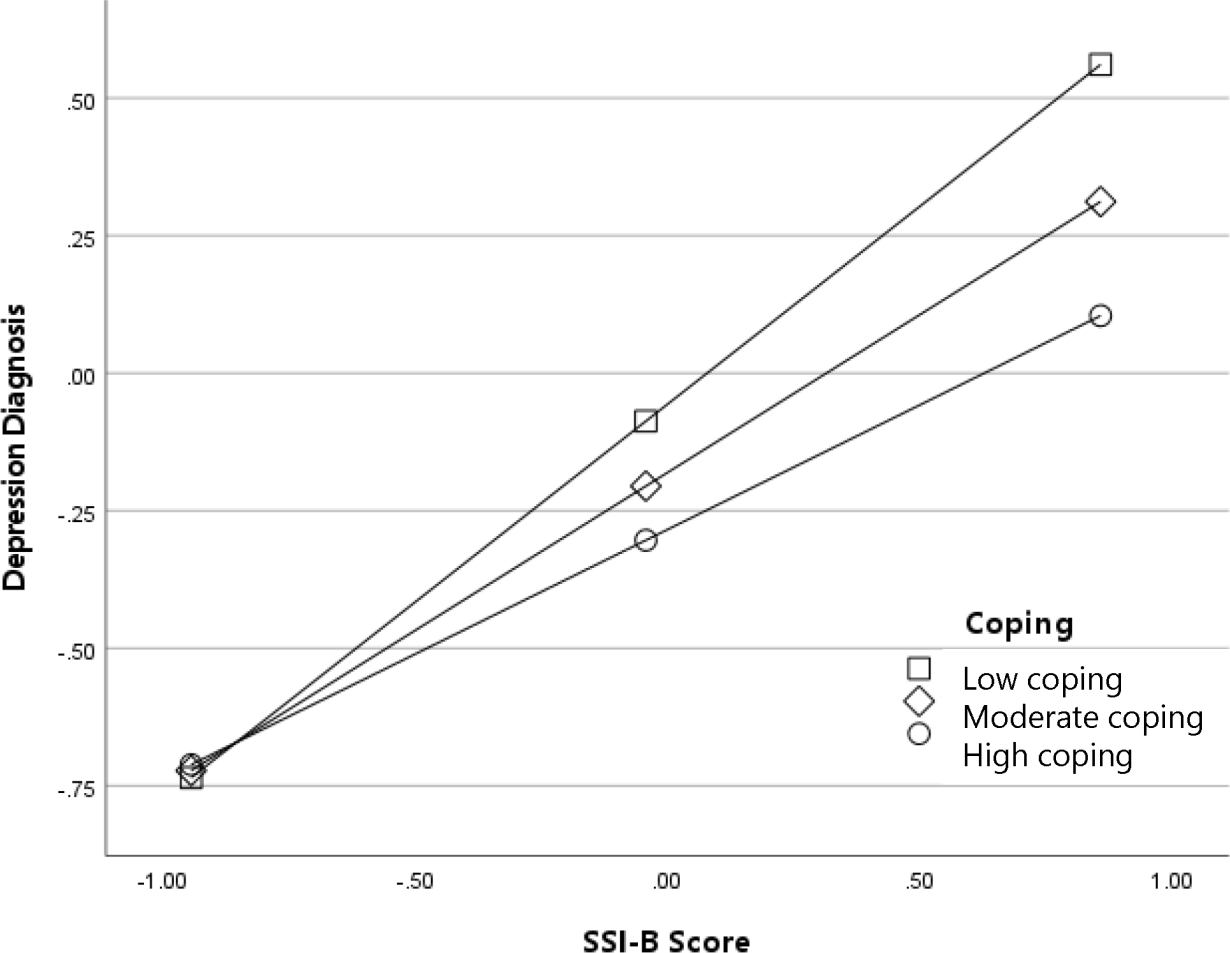
Adaptive coping as a moderator of the association between experienced weight stigma (SSI-B scores) and diagnosis with a depressive disorder (log-odds metric).

To determine the pattern of the interactions, we examined simple effects at high (84% percentile), moderate (50% percentile), and low (14% percentile) values of adaptive coping. Results for the interaction between SSI-B scores (experienced weight stigma) and adaptive coping predicting global mental health are depicted in **Figure 1**. As shown in the figure, the negative association between weight stigma and global mental health was strongest among participants with low frequency of adaptive coping, *B* (95% CI) = -.32 (-.41, -.23), *p* <.001. In contrast, among participants with high frequency of adaptive coping, there was no association between SSI-B scores and global mental health, *B* (95% CI) = -.02 (-.10,.07), *p* = .707. In other words, more frequent engagement in adaptive coping was protective for global mental health even among individuals who experienced weight stigma frequently. Results for the interaction between SSI-B scores and adaptive coping predicting history of a depressive disorder followed a conceptually similar pattern and are depicted in **Figure 2**. The odds of a depressive disorder diagnosis increased with higher exposure to weight stigma; however, the effect was the strongest among participants with low frequency of adaptive coping, log-odds (95% CI) = .72 (.51, .93), OR (95% CI) = 2.05 (1.66, 2.54), *p* <.001, and the weakest among participants with high frequency of adaptive coping, log-odds (95% CI) = .45 (.26, .64), OR (95% CI) = 1.57 (1.30, 1.90), *p* <.001. Thus, again, the odds of receiving a depression diagnosis among participants exposed to weight stigma were lower among individuals who engaged in adaptive coping more frequently.

**Figure 3:**
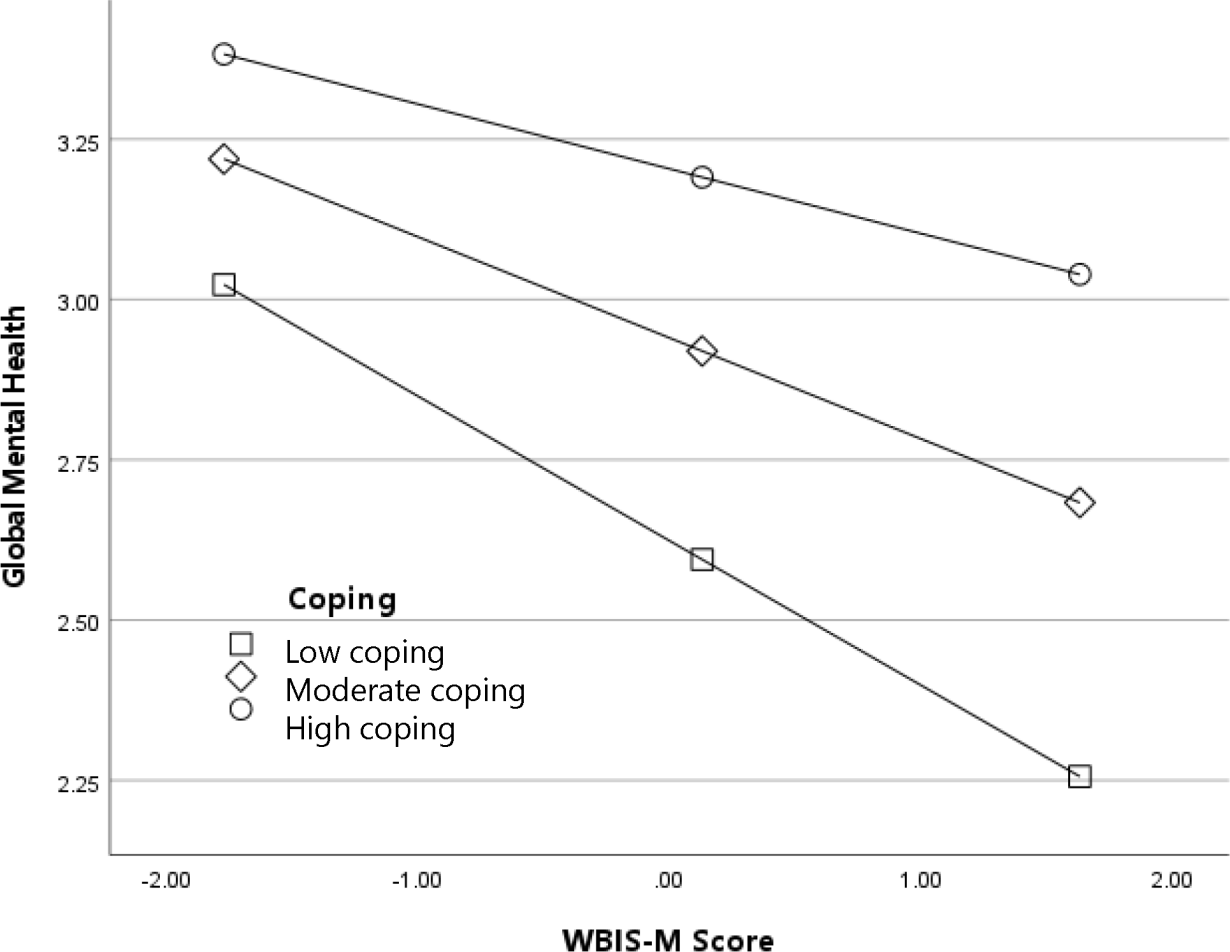
Adaptive coping as a moderator of the association between internalized weight stigma (WBIS-M scores) and global mental health.

**Figure 4:**
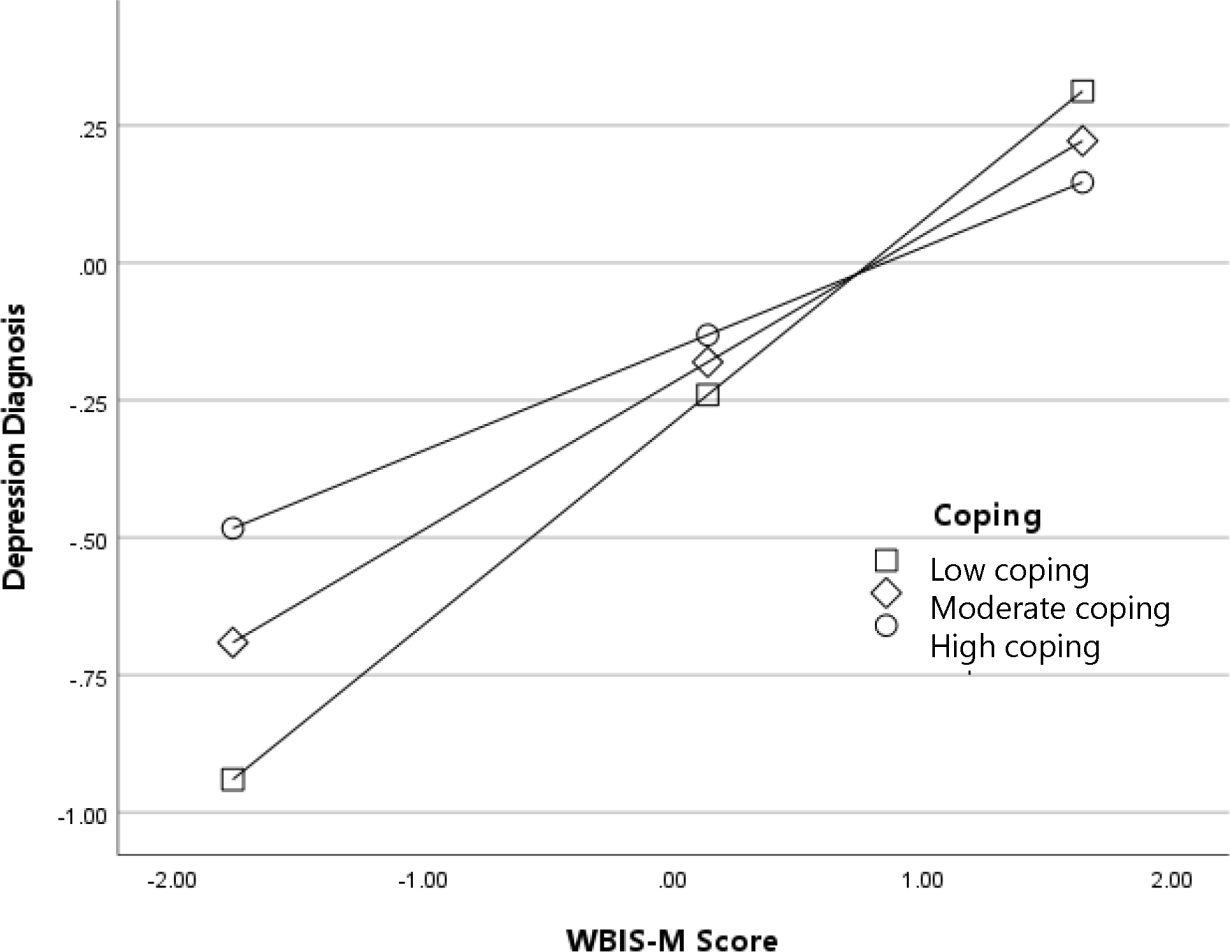
Adaptive coping as a moderator of the association between internalized weight stigma (WBIS-M scores) and diagnosis with a depressive disorder (log-odds metric).

Results for the interaction between WBIS-M scores (internalized weight stigma) and adaptive coping predicting global mental health are depicted in **Figure 3**. In general, participants with higher levels of internalized weight stigma reported worse global mental health; as with the previous analyses, this association was strongest among participants with low frequency of adaptive coping, *B* (95% CI) = -.23 (-.27, -.17), *p* <.001, and weakest among participants with high frequency adaptive coping, *B* (95% CI) = -.10 (-.15, -.06), *p* <.001. Results for the interaction between WBIS-M scores and adaptive coping predicting diagnosis with a depressive disorder are depicted in **Figure 4**. The odds of a diagnosis increased with higher levels of internalized weight stigma; once again, the effect was the strongest among participants with low frequency of adaptive coping, log-odds (95% CI) = .37 (.25, .48), OR (95% CI) = 1.44 (1.29, 1.62), *p* <.001, and the weakest among participants with high frequency of adaptive coping, log-odds (95% CI) = .19 (.09, .29), OR (95% CI) = 1.20 (1.09, 1.33), *p* <.001.

## Discussion

The present study examined the association between weight stigma and three indicators of mental health status in a racially and ethnically diverse sample of adults living in the United States. Consistent with previous research (14), more frequent exposure to weight discrimination and higher levels of internalized weight stigma were associated with poorer global mental health, more severe depressive symptoms, and greater odds of diagnosis with a depressive disorder. Notably, these associations held while controlling for BMI, suggesting that weight stigma confers independent risk to mental health over and above any effects of excess weight itself. Findings suggest that weight stigma is a powerful stressor that may have negative implications for psychological and social wellbeing.

One aim of this study was to assess whether the strength of the association between weight stigma and mental health differs as a function of respondents’ race or ethnicity. Results indicated that neither race nor ethnicity moderated the association between weight stigma and any of the three primary mental health outcomes examined. This finding is consistent with previous meta-analytic research that observed null findings for White versus non-White ethnicity as a moderator of the association between weight stigma and mental health (14). An important limitation of previous work, however, was the limited representation of non-White participants in existing studies, which could reduce the ability to detect such moderating effects.

When interpreting the current null findings, it is important to distinguish between the prevalence of weight stigma (i.e., the extent to which individuals from different subgroups experience or internalize weight stigma) and its association with health outcomes (e.g., the extent to which weight stigma is associated with depression). Some research, for instance, suggests that Black women report relatively fewer experiences with weight discrimination and have lower levels of internalized weight stigma compared to White women (21, 46). Thus, scholars have speculated that Black women may be more protected from the negative health consequences of weight stigma (21). Experiencing weight stigma to a lower degree, however, does not imply that weight stigma, when it does occur, will have lesser consequences. Taken together with previous findings, the current work suggests that although some subgroups may not experience weight stigma quite as often as others do, the implications of weight stigma for mental health are similar among the different racial and ethnic groups examined in the present study. Findings suggest that individuals who experience and/or internalize stigma because of their body weight may be at increased risk for adverse mental health outcomes irrespective of their racial or ethnic identity.

The second aim was to assess factors that may mitigate mental health consequences associated with weight stigma. Results indicated that individuals who use adaptive coping responses to manage weight stigma-related stress may be less vulnerable to poor mental health outcomes. Consistent with hypotheses, more frequent use of adaptive coping was associated with less impaired mental health in the context of weight stigma. It was unclear why adaptive coping moderated effects for global mental health ratings and depressive disorder diagnosis but not severity of depressive symptoms. Emmer and colleagues attempted to examine the moderating effects of adaptive coping in their meta-analysis, yet they were unable to find a sufficient number of studies that had measured adaptive coping strategies and found no studies on social support seeking in particular. More research is needed to identify the primary coping strategies people use to manage weight stigma-related stress and evaluate how coping strategies impact mental health outcomes over time.

Study findings have significant implications for future initiatives aimed at supporting the psychological wellbeing of US adults with high body weight. As long as society continues to denigrate and devalue people because of their weight, it will be important to provide individuals with effective skills to manage weight stigma-related stress and protect their mental wellbeing. Our work suggests the adaptive coping strategies individuals use to manage stress from weight stigma may be protective for mental health. Nevertheless, while the moderating effects of adaptive coping strategies were statistically significant, the practical significance of these effects warrants further examination. Additionally, the observed effect sizes were small, suggesting that while adaptive coping may offer some psychological benefit, it may not fully offset the harmful mental health effects associated with weight stigma. Nonetheless, these findings highlight the potential value of incorporating coping skills—such as cognitive reframing and social support seeking—into future programs designed to mitigate the psychological burdens associated with weight stigma. Future work should use experimental methods to assess which coping strategies are most effective and investigate how promising strategies can be sustainably taught and reinforced in real-world settings. Findings also highlight the need to include participants from diverse racial and ethnic backgrounds in these initiatives, as no subgroup appears protected from the poor mental health consequences associated with weight stigma.

Limitations of the present study provide valuable directions for future research. First, our assessment of global mental health and depression severity relied on validated yet brief instruments—the two-item PROMIS global mental health scale and the PHQ-2. While these measures are widely used for screening purposes and can minimize participant burden, they do not capture the full range of symptoms and functional impairments associated with mental health disorders. Moreover, these tools may overlook important components of psychological wellbeing, such as social connectedness, emotional regulation, and resilience. Future research investigating the association between weight stigma and psychological wellbeing should incorporate more comprehensive measures of mental health. Second, while this study controlled for BMI and several sociodemographic factors, it did not account for other potentially important confounding factors such as stressful life events, comorbid health conditions, other forms of structural and interpersonal discrimination, and socioeconomic stressors. The absence of these variables may have affected the observed results. Future research should incorporate a more comprehensive set of psychosocial and health-related variables to better capture the complex association between weight stigma and mental health. Third, the current study does not offer insight into which types of weight stigma (e.g., interpersonal, environmental, internalized) may be most damaging for mental health. Based on interpersonal theories of depression (47), interpersonal forms of weight stigma could be more consequential, however this remains an empirical question. Fourth, the present findings cannot speak to which coping tactics in particular may be most beneficial for mental wellbeing, nor whether or how coping strategies may vary between different racial/ethnic groups. A related limitation is that although our coping items were informed by our qualitative work and previous research, we did not use an established, validated coping scale. A fifth limitation of this study was the cross-sectional nature of the design thus it is unknown how these processes—both the damaging effects of weight stigma and the protective effects of adaptive coping—unfold over time. Further, we cannot determine whether any of the observed associations are causal. Most studies investigating the association between weight stigma and mental health are cross-sectional, thus there is a significant need for longitudinal and experimental research in this domain.

In closing, this study underscores the significant mental health risks posed by weight stigma, showing that both experienced and internalized stigma are linked to poorer mental wellbeing, including increased depression severity and greater likelihood of diagnosis with a depressive disorder. Notably, these effects were independent of BMI, emphasizing that weight stigma itself may be a harmful stressor beyond effects associated with BMI. Our findings suggest that all individuals, regardless of racial or ethnic identity, may be vulnerable to the detrimental mental health consequences of weight stigma. The study also highlights adaptive coping strategies—such as cognitive reframing and social support seeking—that can buffer individuals from these negative outcomes. Findings suggest that initiatives aimed at promoting adaptive coping could be key in mitigating the psychological impact of weight stigma. Given the pervasive nature of weight bias, it is essential that future initiatives be inclusive of diverse racial and ethnic groups to ensure equitable mental health support for all individuals affected by weight stigma.

## Data Availability

The datasets presented in this study can be found in online repositories. The names of the repository/repositories and accession number(s) can be found below: Open Science Framework https://osf.io/5qsjc/?view_only=9bd700f8afea47cf8cf7ca1cc13574b1.

## References

[1] Puhl RM Heuer CA. The stigma of obesity: a review and update. Obesity. (2009) 17:941–64. doi: 10.1038/oby.2008.636, PMID: 19165161

[2] Major B Tomiyama AJ Hunger J. The negative and bidirectional effects of weight stigma on health. In: Major B Dovidio JF Link BG, editors. The Oxford Handbook of Stigma, Discrimination, and Health, vol. p . Oxford University Press, New York (2018). p. 499–520.

[3] Pearl RL Wadden TA Groshon LC Fitterman-Harris HF Bach C LaFata EM. Refining the conceptualization and assessment of internalized weight stigma: A mixed methods approach. Body Image. (2023) 44:93–102. doi: 10.1016/j.bodyim.2022.12.002, PMID: 36549092

[4] Sutin AR Terracciano A. Sources of weight discrimination and health. Stigma & Health. (2017) 2:23–7. doi: 10.1037/sah0000037, PMID: 28289702 PMC5345569

[5] Gerend MA Patel S Ott N Wetzel K Sutin AR Terracciano A. A qualitative analysis of people’s experiences with weight-based discrimination. Psychol Health. (2022) 37:1093–110. doi: 10.1080/08870446.2021.1921179, PMID: 33979254

[6] Durso LE Latner JD. Understanding self-directed stigma: development of the weight bias internalization scale. Obesity. (2008) 16:S80–S6. doi: 10.1038/oby.2008.448, PMID: 18978768

[7] Lillis J Luoma JB Levin ME Hayes SC. Measuring weight self-stigma: The weight self-stigma questionnaire. Obesity. (2010) 18:971–6. doi: 10.1038/oby.2009.353, PMID: 19834462

[8] Pearl RL Puhl RM. Measuring internalized weight attitudes across body weight categories: validation of the modified weight bias internalization scale. Body Image. (2014) 11:89–92. doi: 10.1016/j.bodyim.2013.09.005, PMID: 24100004

[9] Spahlholz J Baer N Konig HH Riedel-Heller SG Luck-Sikorski C. Obesity and discrimination - A systematic review and meta-analysis of observational studies. Obes Rev. (2016) 17:43–55. doi: 10.1111/obr.12343, PMID: 26596238

[10] Puhl RM Himmelstein MS Quinn DM. Internalizing weight stigma: Prevalence and sociodemographic considerations in US adults. Obesity. (2018) 26:167–75. doi: 10.1002/oby.22029, PMID: 29082666

[11] Pearl RL Puhl RM. Weight bias internalization and health: a systematic review. Obes Rev. (2018) 19:1141–63. doi: 10.1111/obr.12701, PMID: 29788533 PMC6103811

[12] Romano KA Heron KE Sandoval CM MacIntyre RI Howard LM Scott M. Weight bias internalization and psychosocial, physical, and behavioral health: A meta-analysis of cross-sectional and prospective associations. Behav Ther. (2023) 54:539–56. doi: 10.1016/j.beth.2022.12.003, PMID: 37088509 PMC10126478

[13] Wu YK Berry DC. Impact of weight stigma on physiological and psychological health outcomes for overweight and obese adults: A systematic review. J Adv Nursing. (2018) 74:1030–42. doi: 10.1111/jan.13511, PMID: 29171076

[14] Emmer C Bosnjak M Mata J. The association between weight stigma and mental health: A meta-analysis. Obes Rev. (2020) 21:e12935. doi: 10.1111/obr.12935, PMID: 31507062

[15] Pascoe EA Lattanner MR Richman LS. Meta-analysis of interpersonal discrimination and health-related behaviors. Health Psychol. (2022) 41:319–31. doi: 10.1037/hea0001147, PMID: 35467901 PMC11924412

[16] Pascoe EA Richman LS. Perceived discrimination and health: A meta-analytic review. psychol Bull. (2009) 135:531–54. doi: 10.1037/a0016059, PMID: 19586161 PMC2747726

[17] Ratcliffe D Ellison N. Obesity and internalized weight stigma: a formulation model for an emerging psychological problem. Behav Cogn Psychother. (2015) 43:239–52. doi: 10.1017/S1352465813000763, PMID: 25632949

[18] Sikorski C Luppa M Luck T Riedel-Heller SG. Weight stigma “gets under the skin”-evidence for an adapted psychological mediation framework: a systematic review. Obesity. (2015) 23:266–76. doi: 10.1002/oby.20952, PMID: 25627624

[19] Tomiyama AJ. Stress and obesity. Annu Rev Psychol. (2019) 70:703–18. doi: 10.1146/annurev-psych-010418-102936, PMID: 29927688

[20] Hayward LE Vartanian LR Pinkus RT. Coping with weight stigma: development and validation of a Brief Coping Responses Inventory. Obes Sci Pract. (2017) 3:373–83. doi: 10.1002/osp4.125, PMID: 29259795 PMC5729500

[21] Himmelstein MS Puhl RM Quinn DM. Intersectionality: an understudied framework for addressing weight stigma. Am J Prev Med. (2017) 53:421–31. doi: 10.1016/j.amepre.2017.04.003, PMID: 28579331

[22] Himmelstein MS Puhl RM Quinn DM. Weight stigma and health: The mediating role of coping responses. Health Psychol. (2018) 37:139–47. doi: 10.1037/hea0000575, PMID: 29120192

[23] Major B Hunger JM Bunyan DP Miller CT. The ironic effects of weight stigma. J Exp Soc Psychol. (2014) 51:74–80. doi: 10.1016/j.jesp.2013.11.009

[24] Schvey NA Puhl RM Brownell KD. The impact of weight stigma on caloric consumption. Obes (Silver Spring). (2011) 19:1957–62. doi: 10.1038/oby.2011.204, PMID: 21760636

[25] Gerend MA Zetrenne S Sutin AR Naar S Maner JK. Weight discrimination and health risk behavior in racial, ethnic, and sexual minority adults. Ann Behav Med. (2023) 57:571–81. doi: 10.1093/abm/kaad003, PMID: 37061832

[26] Lee KM Hunger JM Tomiyama AJ. Weight stigma and health behaviors: evidence from the Eating in America Study. Int J Obes. (2021) 45:1499–509. doi: 10.1038/s41366-021-00814-5, PMID: 33934109 PMC8236399

[27] Tomiyama AJ. Weight stigma is stressful. a review of evidence for the Cyclic Obesity/Weight-Based Stigma model. Appetite. (2014) 82:8–15. doi: 10.1016/j.appet.2014.06.108, PMID: 24997407

[28] Gerend MA Patel S Ott N Wetzel K Sutin AR Terracciano A. Coping with weight discrimination: Findings from a qualitative study. Stigma Health. (2021) 6:440–9. doi: 10.1037/sah0000335

[29] Himmelstein MS Incollingo Belsky AC Tomiyama AJ. The weight of stigma: Cortisol reactivity to manipulated weight stigma. Obesity. (2015) 23:368–74. doi: 10.1002/oby.20959, PMID: 25522347

[30] Hunger JM Blodorn A Miller CT Major B. The psychological and physiological effects of interacting with an anti-fat peer. Body Image. (2018) 27:148–55. doi: 10.1016/j.bodyim.2018.09.002, PMID: 30267954

[31] Schvey NA Puhl RM Brownell KD. The stress of stigma: Exploring the effect of weight stigma on cortisol reactivity. Psychosom Med. (2014) 76:156–62. doi: 10.1097/PSY.0000000000000031, PMID: 24434951

[32] Sutin AR Stephan Y Luchetti M Terracciano A. Perceived weight discrimination and C-reactive protein. Obes (Silver Spring). (2014) 22:1959–61. doi: 10.1002/oby.20789, PMID: 24828961

[33] Lewis TT Cogburn CD Williams DR. Self-reported experiences of discrimination and health: scientific advances, ongoing controversies, and emerging issues. Annu Rev Clin Psychol. (2015) 11:407–40. doi: 10.1146/annurev-clinpsy-032814-112728, PMID: 25581238 PMC5555118

[34] Williams DR Mohammed SA. Discrimination and racial disparities in health: Evidence and needed research. J Behav Med. (2009) 32:20–47. doi: 10.1007/s10865-008-9185-0, PMID: 19030981 PMC2821669

[35] Gerend MA Stewart C Wetzel K. Vulnerability and resilience to the harmful health consequences of weight discrimination in Black, Latina, and sexual minority women. Soc Sci Med. (2022) 315:115555. doi: 10.1016/j.socscimed.2022.115555, PMID: 36423540

[36] Himmelstein MS Puhl RM Pearl RL Pinto AM Foster GD. Coping with weight stigma among adults in a commercial weight management sample. Int J Behav Med. (2020) 27:576–90. doi: 10.1007/s12529-020-09895-4, PMID: 32430784

[37] Vartanian LR. Development and validation of a brief version of the Stigmatizing Situations Inventory. Obes Sci Pract. (2015) 1:119–25. doi: 10.1002/osp4.11, PMID: 27774254 PMC5063154

[38] Myers A Rosen JC. Obesity stigmatization and coping: relation to mental health symptoms, body image, and self-esteem. Int J Obes Relat Metab Disord. (1999) 23:221–30. doi: 10.1038/sj.ijo.0800765, PMID: 10193866

[39] Puhl RM Brownell KD. Confronting and coping with weight stigma: an investigation of overweight and obese adults. Obesity. (2006) 14:1802–15. doi: 10.1038/oby.2006.208, PMID: 17062811

[40] Levy M Kakinami L Alberga AS. The relationship between weight bias internalization and healthy and unhealthy weight control behaviours. Eating Weight Disord. (2022) 27:1621–32. doi: 10.1007/s40519-021-01291-5, PMID: 35201546

[41] Hays RD Schalet BD Spritzer KL Cella D. Two-item PROMIS(R) global physical and mental health scales. J Patient Rep Outcomes. (2017) 1:2. doi: 10.1186/s41687-017-0003-8, PMID: 29757325 PMC5934936

[42] Lowe B Kroenke K Grafe K. Detecting and monitoring depression with a two-item questionnaire (PHQ-2). J Psychosom Res. (2005) 58:163–71. doi: 10.1016/j.jpsychores.2004.09.006, PMID: 15820844

[43] Centers for Disease Control and Prevention. Behavioral Risk Factor Surveillance System Survey Questionnaire. Atlanta, Georgia: U.S. Department of Health and Human Services, Centers for Disease Control and Prevention (2020).

[44] McNeilly MD Anderson NB Armstead CA Clark R Corbett M Robinson EL. The perceived racism scale: a multidimensional assessment of the experience of white racism among African Americans. Ethn Dis. (1996) 6:154–66., PMID: 8882844

[45] van Buuren S. Flexible Imputation of Missing Data. 2nd ed. New York: Chapman and Hall/CRC (2018).

[46] Dutton GR Lewis TT Durant N Halanych J Kiefe CI Sidney S. Perceived weight discrimination in the CARDIA study: differences by race, sex, and weight status. Obesity. (2014) 22:530–6. doi: 10.1002/oby.20438, PMID: 23512948 PMC3695009

[47] Joiner TE Van Orden KA Witte TK Selby EA Ribeiro JD Lewis R. Main predictions of the interpersonal-psychological theory of suicidal behavior: empirical tests in two samples of young adults. J Abnorm Psychol. (2009) 118:634–46. doi: 10.1037/a0016500, PMID: 19685959 PMC2846517

