# Supplementary material for: Weight stigma and mental health in a racially and ethnically diverse sample of US adults
Source: Front Psychiatry. 2025 Jul 21;16:1593145. doi: 10.3389/fpsyt.2025.1593145 (PMC12319583; doi:10.3389/fpsyt.2025.1593145)
Supplement: Supplementary file 1 [file Supplementaryfile1.docx]

Supplemental Material

Weight Stigma and Mental Health in a Racially and Ethnically Diverse Sample of US Adults

Frontiers in Psychiatry

Results from Exploratory Factor Analysis for Coping Strategies

FACTOR

/VARIABLES Q65_1 Q65_2 Q65_3 Q65_4 Q65_5 Q65_6 Q65_7 Q65_8 Q65_9 Q65_10 Q65_11 Q65_12 Q65_13

Q65_14 Q65_15 Q65_16 Q65_17

/MISSING LISTWISE

/ANALYSIS Q65_1 Q65_2 Q65_3 Q65_4 Q65_5 Q65_6 Q65_7 Q65_8 Q65_9 Q65_10 Q65_11 Q65_12 Q65_13

Q65_14 Q65_15 Q65_16 Q65_17

/PRINT INITIAL EXTRACTION ROTATION

/PLOT EIGEN

/CRITERIA MINEIGEN(1) ITERATE(25)

/EXTRACTION PAF

/CRITERIA ITERATE(25) DELTA(0)

/ROTATION OBLIMIN

/METHOD=CORRELATION.

- - - - - - - - - - - - - - - - - - - - - - - - F A C T O R A N A L Y S I S - - - - - - - - - - - - - - - - - - - - - - - -

**Factor Analysis**

| **Communalities** | | |
| --- | --- | --- |
|  | Initial | Extraction |
| Q65_1 When you are teased, treated unfairly, or discriminated against because of your weight, how often do you do any of the following things in response? - Talk to other people about it | .301 | .372 |
| Q65_2 When you are teased, treated unfairly, or discriminated against because of your weight, how often do you do any of the following things in response? - Eat | .320 | .363 |
| Q65_3 When you are teased, treated unfairly, or discriminated against because of your weight, how often do you do any of the following things in response? - Do something to distract yourself | .354 | .403 |
| Q65_4 When you are teased, treated unfairly, or discriminated against because of your weight, how often do you do any of the following things in response? - Blame yourself for what happened | .486 | .572 |
| Q65_5 When you are teased, treated unfairly, or discriminated against because of your weight, how often do you do any of the following things in response? - Pray about the situation | .280 | .317 |
| Q65_6 When you are teased, treated unfairly, or discriminated against because of your weight, how often do you do any of the following things in response? - Avoid the person, place, or situation in the future | .388 | .422 |
| Q65_7 When you are teased, treated unfairly, or discriminated against because of your weight, how often do you do any of the following things in response? - Speak up for yourself | .382 | .448 |
| Q65_8 When you are teased, treated unfairly, or discriminated against because of your weight, how often do you do any of the following things in response? - See it as their problem, not yours | .365 | .414 |
| Q65_9 When you are teased, treated unfairly, or discriminated against because of your weight, how often do you do any of the following things in response? - Express anger or get mad | .349 | .379 |
| Q65_10 When you are teased, treated unfairly, or discriminated against because of your weight, how often do you do any of the following things in response? - Work harder to prove them wrong | .324 | .349 |
| Q65_11 When you are teased, treated unfairly, or discriminated against because of your weight, how often do you do any of the following things in response? - Think bad thoughts about yourself | .534 | .622 |
| Q65_12 When you are teased, treated unfairly, or discriminated against because of your weight, how often do you do any of the following things in response? - Withdraw socially | .489 | .565 |
| Q65_13 When you are teased, treated unfairly, or discriminated against because of your weight, how often do you do any of the following things in response? - Think about your good qualities | .490 | .576 |
| Q65_14 When you are teased, treated unfairly, or discriminated against because of your weight, how often do you do any of the following things in response? - Ignore them and try not to let them get to you | .315 | .421 |
| Q65_15 When you are teased, treated unfairly, or discriminated against because of your weight, how often do you do any of the following things in response? - Love and accept yourself, even when it seems like other people don’t | .502 | .611 |
| Q65_16 When you are teased, treated unfairly, or discriminated against because of your weight, how often do you do any of the following things in response? - Use humor to defuse the situation | .254 | .267 |
| Q65_17 When you are teased, treated unfairly, or discriminated against because of your weight, how often do you do any of the following things in response? - Pretend not to care | .326 | .442 |
| Extraction Method: Principal Axis Factoring. | | |

| **Total Variance Explained** | | | | | | | |
| --- | --- | --- | --- | --- | --- | --- | --- |
| Factor | Initial Eigenvalues | | | Extraction Sums of Squared Loadings | | | Rotation Sums of Squared Loadings^a^ |
|  | Total | % of Variance | Cumulative % | Total | % of Variance | Cumulative % | Total |
| 1 | 5.087 | 29.922 | 29.922 | 4.523 | 26.607 | 26.607 | 3.538 |
| 2 | 2.866 | 16.862 | 46.784 | 2.402 | 14.129 | 40.736 | 3.375 |
| 3 | 1.213 | 7.138 | 53.922 | .620 | 3.644 | 44.380 | 2.226 |
| 4 | .813 | 4.781 | 58.703 |  |  |  |  |
| 5 | .794 | 4.673 | 63.376 |  |  |  |  |
| 6 | .704 | 4.142 | 67.519 |  |  |  |  |
| 7 | .648 | 3.809 | 71.328 |  |  |  |  |
| 8 | .609 | 3.585 | 74.912 |  |  |  |  |
| 9 | .576 | 3.389 | 78.301 |  |  |  |  |
| 10 | .551 | 3.244 | 81.545 |  |  |  |  |
| 11 | .539 | 3.170 | 84.715 |  |  |  |  |
| 12 | .520 | 3.059 | 87.774 |  |  |  |  |
| 13 | .468 | 2.753 | 90.526 |  |  |  |  |
| 14 | .461 | 2.713 | 93.239 |  |  |  |  |
| 15 | .425 | 2.500 | 95.739 |  |  |  |  |
| 16 | .377 | 2.219 | 97.958 |  |  |  |  |
| 17 | .347 | 2.042 | 100.000 |  |  |  |  |
| Extraction Method: Principal Axis Factoring. | | | | | | | |
| a. When factors are correlated, sums of squared loadings cannot be added to obtain a total variance. | | | | | | | |


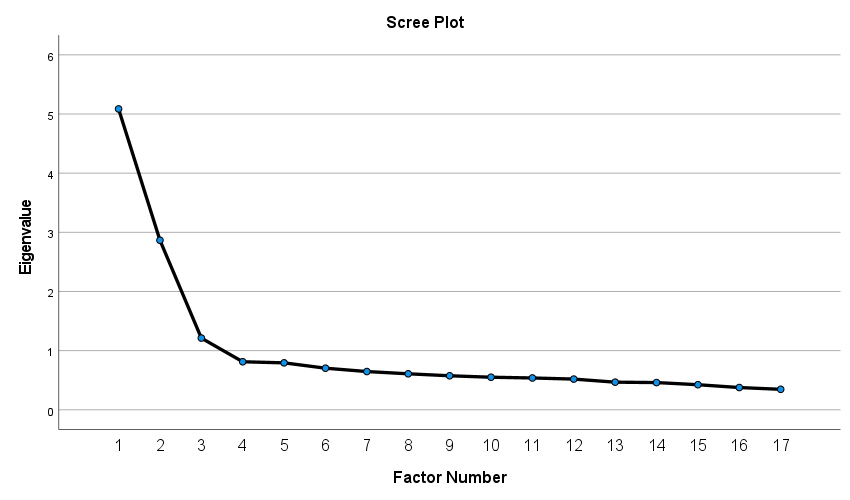


| **Factor Matrix^a^** | | | |
| --- | --- | --- | --- |
|  | Factor | | |
|  | 1 | 2 | 3 |
| Q65_1 When you are teased, treated unfairly, or discriminated against because of your weight, how often do you do any of the following things in response? - Talk to other people about it | .530 | .144 | .266 |
| Q65_2 When you are teased, treated unfairly, or discriminated against because of your weight, how often do you do any of the following things in response? - Eat | .532 | -.201 | .201 |
| Q65_3 When you are teased, treated unfairly, or discriminated against because of your weight, how often do you do any of the following things in response? - Do something to distract yourself | .612 | -.054 | -.161 |
| Q65_4 When you are teased, treated unfairly, or discriminated against because of your weight, how often do you do any of the following things in response? - Blame yourself for what happened | .516 | -.538 | .127 |
| Q65_5 When you are teased, treated unfairly, or discriminated against because of your weight, how often do you do any of the following things in response? - Pray about the situation | .495 | .100 | .248 |
| Q65_6 When you are teased, treated unfairly, or discriminated against because of your weight, how often do you do any of the following things in response? - Avoid the person, place, or situation in the future | .566 | -.282 | -.151 |
| Q65_7 When you are teased, treated unfairly, or discriminated against because of your weight, how often do you do any of the following things in response? - Speak up for yourself | .459 | .449 | .189 |
| Q65_8 When you are teased, treated unfairly, or discriminated against because of your weight, how often do you do any of the following things in response? - See it as their problem, not yours | .426 | .482 | .008 |
| Q65_9 When you are teased, treated unfairly, or discriminated against because of your weight, how often do you do any of the following things in response? - Express anger or get mad | .535 | -.232 | .198 |
| Q65_10 When you are teased, treated unfairly, or discriminated against because of your weight, how often do you do any of the following things in response? - Work harder to prove them wrong | .560 | .159 | .102 |
| Q65_11 When you are teased, treated unfairly, or discriminated against because of your weight, how often do you do any of the following things in response? - Think bad thoughts about yourself | .512 | -.600 | .002 |
| Q65_12 When you are teased, treated unfairly, or discriminated against because of your weight, how often do you do any of the following things in response? - Withdraw socially | .572 | -.476 | -.110 |
| Q65_13 When you are teased, treated unfairly, or discriminated against because of your weight, how often do you do any of the following things in response? - Think about your good qualities | .495 | .576 | .004 |
| Q65_14 When you are teased, treated unfairly, or discriminated against because of your weight, how often do you do any of the following things in response? - Ignore them and try not to let them get to you | .483 | .249 | -.354 |
| Q65_15 When you are teased, treated unfairly, or discriminated against because of your weight, how often do you do any of the following things in response? - Love and accept yourself, even when it seems like other people don’t | .383 | .677 | -.078 |
| Q65_16 When you are teased, treated unfairly, or discriminated against because of your weight, how often do you do any of the following things in response? - Use humor to defuse the situation | .508 | .056 | -.074 |
| Q65_17 When you are teased, treated unfairly, or discriminated against because of your weight, how often do you do any of the following things in response? - Pretend not to care | .538 | -.071 | -.384 |
| Extraction Method: Principal Axis Factoring. | | | |
| a. 3 factors extracted. 6 iterations required. | | | |

| **Pattern Matrix^a^** | | | |
| --- | --- | --- | --- |
|  | Factor | | |
|  | 1 | 2 | 3 |
| Q65_1 When you are teased, treated unfairly, or discriminated against because of your weight, how often do you do any of the following things in response? - Talk to other people about it | .293 | .519 | .099 |
| Q65_2 When you are teased, treated unfairly, or discriminated against because of your weight, how often do you do any of the following things in response? - Eat | .539 | .216 | .048 |
| Q65_3 When you are teased, treated unfairly, or discriminated against because of your weight, how often do you do any of the following things in response? - Do something to distract yourself | .337 | .178 | -.360 |
| Q65_4 When you are teased, treated unfairly, or discriminated against because of your weight, how often do you do any of the following things in response? - Blame yourself for what happened | .767 | -.093 | -.006 |
| Q65_5 When you are teased, treated unfairly, or discriminated against because of your weight, how often do you do any of the following things in response? - Pray about the situation | .301 | .457 | .093 |
| Q65_6 When you are teased, treated unfairly, or discriminated against because of your weight, how often do you do any of the following things in response? - Avoid the person, place, or situation in the future | .492 | -.017 | -.323 |
| Q65_7 When you are teased, treated unfairly, or discriminated against because of your weight, how often do you do any of the following things in response? - Speak up for yourself | -.015 | .680 | .026 |
| Q65_8 When you are teased, treated unfairly, or discriminated against because of your weight, how often do you do any of the following things in response? - See it as their problem, not yours | -.127 | .593 | -.153 |
| Q65_9 When you are teased, treated unfairly, or discriminated against because of your weight, how often do you do any of the following things in response? - Express anger or get mad | .565 | .192 | .045 |
| Q65_10 When you are teased, treated unfairly, or discriminated against because of your weight, how often do you do any of the following things in response? - Work harder to prove them wrong | .239 | .458 | -.082 |
| Q65_11 When you are teased, treated unfairly, or discriminated against because of your weight, how often do you do any of the following things in response? - Think bad thoughts about yourself | .767 | -.211 | -.131 |
| Q65_12 When you are teased, treated unfairly, or discriminated against because of your weight, how often do you do any of the following things in response? - Withdraw socially | .662 | -.143 | -.273 |
| Q65_13 When you are teased, treated unfairly, or discriminated against because of your weight, how often do you do any of the following things in response? - Think about your good qualities | -.161 | .699 | -.183 |
| Q65_14 When you are teased, treated unfairly, or discriminated against because of your weight, how often do you do any of the following things in response? - Ignore them and try not to let them get to you | -.046 | .248 | -.535 |
| Q65_15 When you are teased, treated unfairly, or discriminated against because of your weight, how often do you do any of the following things in response? - Love and accept yourself, even when it seems like other people don’t | -.336 | .678 | -.237 |
| Q65_16 When you are teased, treated unfairly, or discriminated against because of your weight, how often do you do any of the following things in response? - Use humor to defuse the situation | .223 | .258 | -.242 |
| Q65_17 When you are teased, treated unfairly, or discriminated against because of your weight, how often do you do any of the following things in response? - Pretend not to care | .225 | .010 | -.567 |
| Extraction Method: Principal Axis Factoring.  Rotation Method: Oblimin with Kaiser Normalization. | | | |
| a. Rotation converged in 25 iterations. | | | |

| **Structure Matrix** | | | |
| --- | --- | --- | --- |
|  | Factor | | |
|  | 1 | 2 | 3 |
| Q65_1 When you are teased, treated unfairly, or discriminated against because of your weight, how often do you do any of the following things in response? - Talk to other people about it | .364 | .540 | -.151 |
| Q65_2 When you are teased, treated unfairly, or discriminated against because of your weight, how often do you do any of the following things in response? - Eat | .567 | .301 | -.163 |
| Q65_3 When you are teased, treated unfairly, or discriminated against because of your weight, how often do you do any of the following things in response? - Do something to distract yourself | .463 | .362 | -.507 |
| Q65_4 When you are teased, treated unfairly, or discriminated against because of your weight, how often do you do any of the following things in response? - Blame yourself for what happened | .751 | .052 | -.172 |
| Q65_5 When you are teased, treated unfairly, or discriminated against because of your weight, how often do you do any of the following things in response? - Pray about the situation | .362 | .482 | -.138 |
| Q65_6 When you are teased, treated unfairly, or discriminated against because of your weight, how often do you do any of the following things in response? - Avoid the person, place, or situation in the future | .572 | .183 | -.444 |
| Q65_7 When you are teased, treated unfairly, or discriminated against because of your weight, how often do you do any of the following things in response? - Speak up for yourself | .105 | .668 | -.198 |
| Q65_8 When you are teased, treated unfairly, or discriminated against because of your weight, how often do you do any of the following things in response? - See it as their problem, not yours | .023 | .620 | -.319 |
| Q65_9 When you are teased, treated unfairly, or discriminated against because of your weight, how often do you do any of the following things in response? - Express anger or get mad | .589 | .282 | -.164 |
| Q65_10 When you are teased, treated unfairly, or discriminated against because of your weight, how often do you do any of the following things in response? - Work harder to prove them wrong | .345 | .530 | -.297 |
| Q65_11 When you are teased, treated unfairly, or discriminated against because of your weight, how often do you do any of the following things in response? - Think bad thoughts about yourself | .761 | -.024 | -.258 |
| Q65_12 When you are teased, treated unfairly, or discriminated against because of your weight, how often do you do any of the following things in response? - Withdraw socially | .706 | .072 | -.396 |
| Q65_13 When you are teased, treated unfairly, or discriminated against because of your weight, how often do you do any of the following things in response? - Think about your good qualities | .016 | .730 | -.376 |
| Q65_14 When you are teased, treated unfairly, or discriminated against because of your weight, how often do you do any of the following things in response? - Ignore them and try not to let them get to you | .138 | .418 | -.606 |
| Q65_15 When you are teased, treated unfairly, or discriminated against because of your weight, how often do you do any of the following things in response? - Love and accept yourself, even when it seems like other people don’t | -.149 | .695 | -.378 |
| Q65_16 When you are teased, treated unfairly, or discriminated against because of your weight, how often do you do any of the following things in response? - Use humor to defuse the situation | .334 | .381 | -.386 |
| Q65_17 When you are teased, treated unfairly, or discriminated against because of your weight, how often do you do any of the following things in response? - Pretend not to care | .372 | .242 | -.628 |
| Extraction Method: Principal Axis Factoring.  Rotation Method: Oblimin with Kaiser Normalization. | | | |

| **Factor Correlation Matrix** | | | |
| --- | --- | --- | --- |
| Factor | 1 | 2 | 3 |
| 1 | 1.000 | .186 | -.257 |
| 2 | .186 | 1.000 | -.335 |
| 3 | -.257 | -.335 | 1.000 |
| Extraction Method: Principal Axis Factoring.  Rotation Method: Oblimin with Kaiser Normalization. | | | |
